# The elemental defense effect of cadmium on *Alternaria brassicicola* in *Brassica juncea*

**DOI:** 10.1186/s12870-021-03398-4

**Published:** 2022-01-05

**Authors:** Zhe Liu, Zhenzhen Sun, Chaozhen Zeng, Xujie Dong, Mei Li, Zhixiang Liu, Mingli Yan

**Affiliations:** 1 Hunan Provincial Key Laboratory of Forestry Biotechnology, College of Life Science and Technology, Central South University of Forestry and Technology, Changsha, 410004 China; 2 International Cooperation Base of Science and Technology Innovation on Forest Resource Biotechnology of Hunan Province, Central South University of Forestry and Technology, Changsha, 410004 China; 3 Hunan Provincial Key Laboratory of Crop Germplasm Innovation and Utilization, Hunan Agricultural University, Changsha, 410128 China; 4 Crop Research Institute, Hunan Academy of Agricultural Sciences, Changsha, 410125 China; 5 Hunan Key Laboratory of Economic Crops Genetic Improvement and Integrated Utilization, Hunan University of Science and Technology, Xiangtan, 411201 China

**Keywords:** MiRNAs, Systemic acquired resistance (SAR), Defense-related genes, Cadmium

## Abstract

**Background:**

The elemental defense hypothesis states a new defensive strategy that hyperaccumulators defense against herbivores or pathogens attacks by accumulating heavy metals. *Brassica juncea* has an excellent ability of cadmium (Cd) accumulation. However, the elemental defense effect and its regulation mechanism in *B. juncea* remain unclear.

**Results:**

In this study, we profiled the elemental defense effect and the molecular regulatory mechanism in Cd-accumulated *B. juncea* after *Alternaria brassicicola* infection. *B. juncea* treated with 180 mg Kg^− 1^ DW CdCl_2_ 2.5H_2_O exhibited obvious elemental defense effect after 72 h of infection with *A. brassicicola*. The expression of some defense-related genes including *BjNPR1*, *BjPR12, BjPR2*, and stress-related miRNAs (miR156, miR397, miR398a, miR398b/c, miR408, miR395a, miR395b, miR396a, and miR396b) were remarkably elevated during elemental defense in *B. juncea*.

**Conclusions:**

The results indicate that Cd-accumulated *B. juncea* may defend against pathogens by coordinating salicylic acid (SA) and jasmonic acid (JA) mediated systemic acquired resistance (SAR) and elemental defense in a synergistic joint effect. Furthermore, the expression of miRNAs related to heavy metal stress response and disease resistance may regulate the balance between pathogen defense and heavy metal stress-responsive in *B. juncea*. The findings provide experimental evidence for the elemental defense hypothesis in plants from the perspectives of phytohormones, defense-related genes, and miRNAs.

**Supplementary Information:**

The online version contains supplementary material available at 10.1186/s12870-021-03398-4.

## Background

The term “heavy metals” refers to a series of metals and metalloids that can be toxic to plants and animals at very low content, such as cadmium (Cd), arsenic(As), cobalt (Co), copper (Cu), manganese (Mn), nickel (Ni), lead (Pb), stibium (Sb), selenium (Se), thallium (Tl), zinc (Zn) [1]. Hyperaccumulators can survive in soils with high concentrations of heavy metals and accumulate heavy metals concentration at hundreds or even thousands of times more than non-hyperaccumulator plants [2]. To explain the adaptive significance of accumulated heavy metals to plants on evolution, the elemental defense hypothesis has been proposed and supported by much experimental evidence [3]. This hypothesis believes that some plants can utilize the accumulated heavy metals to defend against pathogens or herbivores, thereby gaining growth and development benefits [4]. There are early reports supporting this hypothesis: the bacterium *Xanthomonas campestris* and the fungus *Alternaria brassicicola* in *Streptanthus polygaloides*, as well as the genus *Pythium* in *Alyssum* species, were significantly inhibited by nickel (Ni) [5, 6], whereas selenium defends *Brassica juncea* against *Alternaria brassicicola and Fusarium* [7]. Reduced growth or survival of *Pseudomonas syringae* and *Tribolium confusum* by the direct inhibition from Ni in a concentration-dependent manner was recorded in herbaceous plant *Thlaspi caerulescens* and *Streptanthus polygaloides* seeds, respectively [8, 9]. The elemental defense effect has also been determined in woody plants. Leaf lesion area of Cd-accumulated *Populus yunnanensis* decreased 37% compared with untreated-Cd samples when challenged by pathogenic fungus *Pestalotiopsis microspora* [4]. Additionally, Boyd [10] believes that a heavy metal element is combined with other defense chemicals to create an additive or synergistic effect, and the heavy metal can exert protective effects on plants at much lower concentrations.

The defensive model was considered to be a brand new strategy that works differently from other forms of defense [11]. The evidence shows that the defensive behavior can be produced in concert with organic defense and participate in defensive response mutually, and this cooperation refers to a trade-off [12]. Plants defend against natural enemies by heavy metals, thereby reducing their dependence on organic defense. It has been reported that the amount of camalexin induction was reduced under elemental defense [13], which suggests that elemental defense could replace the organic defense to a certain extent. The systemic acquired resistance (SAR), an inducible plant immune response, is a key defense process against pathogens in plants [14]. SAR has two obvious characteristics: (1) SAR is closely related to pathogenesis-related genes (PRs). It can even be said that SAR is a result of the expression of PR genes. *PR1*, *PR2*, *PR3*, *PR5*, and plant defensin (*PDF12*) were used as markers for SAR *in Brassica juncea* [15, 16]. (2) SAR is usually accompanied by an accumulation of salicylic acid (SA) [17]. Conversely, reducing SA accumulation weakens the strength of SAR and the expression on related PR genes [18, 19]. These suggest that SA mediates a key plant defense mode (SAR), in which PR genes play important roles. The reduction of SA and enhancement of jasmonic acid (JA) were found in *Noccaea praecox* leaves after Cd accumulation under the pathogen attack. JA-mediated defense pathways may be selected to deal with more biotic stresses in *N. praecox* accumulated heavy metals [20]. Cross talk between SA and JA mediated signaling was also reported in former works [21], SA pathway-related PR genes could be induced by JA, and JA-induced PR genes may also be up-regulated under SA accumulation [16]. Furthermore, under Cd stress, stress-related plant hormones, including abscisic acid (ABA), indole acetic acid (IAA), SA, and JA, were significantly changed, and the expression of phytohormones synthesis related genes were also greatly affected [22–24].

At present, the possible mechanism of the hypothesis has also been reported. Cd-induced resistance to *Fusarium oxysporum* is closely related to metal-induced proteins in wheat [25]. Metal ions, such as mercury (Hg), cause membrane damage and were regarded as elicitors of defense compounds [26]. In addition, they can activate effectively defense signaling pathways [20, 27]. There may be synergism and trade-offs between metal accumulation and the salicylate and/or ROS signaling in elemental defense effect [28]. In general, metal accumulation and plant hormone signaling pathways were hired in anti-pathogen responses in Brassicaceae, but the elemental defense, SA induced SAR and the trade-off between them remain unclear.

Plant miRNAs belong to short non-coding RNAs, most of which are 20–24 nt in length and participate in the regulation of plant development, biotic and abiotic stress response, and other physiological processes by regulating the expression of their target genes [29, 30].

A variety of miRNAs and their targets related to heavy metal stress responses have been reported in plants [31–33]. For example, Zhou, et al. [32] reported that miR156 targets the transcript of glutathione-γ-glutamylcysteinyl transferase (GGT) in *Brassica napus* under Cd stress. GGT, as well as phytochelatin synthase, constitutes the main mechanisms of heavy metal detoxification. MiR397 played a crucial role in the tolerance to Boron toxicity by targeting Laccase (*LAC*) genes in *Citrus* [34]. Furthermore, miR408 regulated Cd tolerance by targeting ascorbate oxidase in soybean [35]. The accumulated evidence suggests that miR398 mainly targets Cu/Zn superoxide dismutases (CSD), which is the main superoxide dismutase in plant resistance to reactive oxygen species (ROS) toxicity by affecting various abiotic stresses, such as high light, heavy metals, drought, and oxidative stress [36, 37].

There are also many miRNAs associated with disease resistance. For instance, miR393 was involved in the defense against *Phytophthora sojae* in soybean [38]. MiR395 mainly targets the regulation of the sulfate transporter (SULTR2) and three genes of the ATP sulfurylase (ATPS) gene family, which can assimilate sulfate to form glutathione for providing antioxidant defense against pathogen induced oxygen free radical mediated damage [39]. MiR396 is involved in dynamic defense response against both necrotrophic and hemibiotrophic fungal pathogens [40].

*B. juncea* can be used as a phytoremediation material for heavy metal pollution of soil because of its strong enrichment and tolerance to Cd [41]. *Alternaria brassicicola* can cause *Alternaria* rot in a variety of *Brassica* plants, such as *Brassica rapa* and *Brassica oleracea*, thereby causing serious economic losses [42]. The purpose of this study was to investigate the elemental defense effect of Cd in *B. juncea* after *A. brassicicola* infection, providing new evidence for the elemental defense hypothesis from the perspectives of phytohormones, PR genes, and miRNAs.

## Results

### Pathogenic effect of *A. brassicicola* in *B. juncea* leaves

As illustrated in Fig. S1, the disease spot parameters, such as diameter, area, and perimeter were calculated by Image J software. The disease spots were more obvious at 72 hpi. With the extension of time, the hyphae of *A. brassicicola* were widened and showed obvious segmental diaphragm at 72 hpi (Fig. S2).

### The elemental defense effect of *B. juncea* enriched with Cd against *A. brassicicola*

#### In vitro toxicity of Cd to *A*. *brassicicola*

**Fig. 1:**
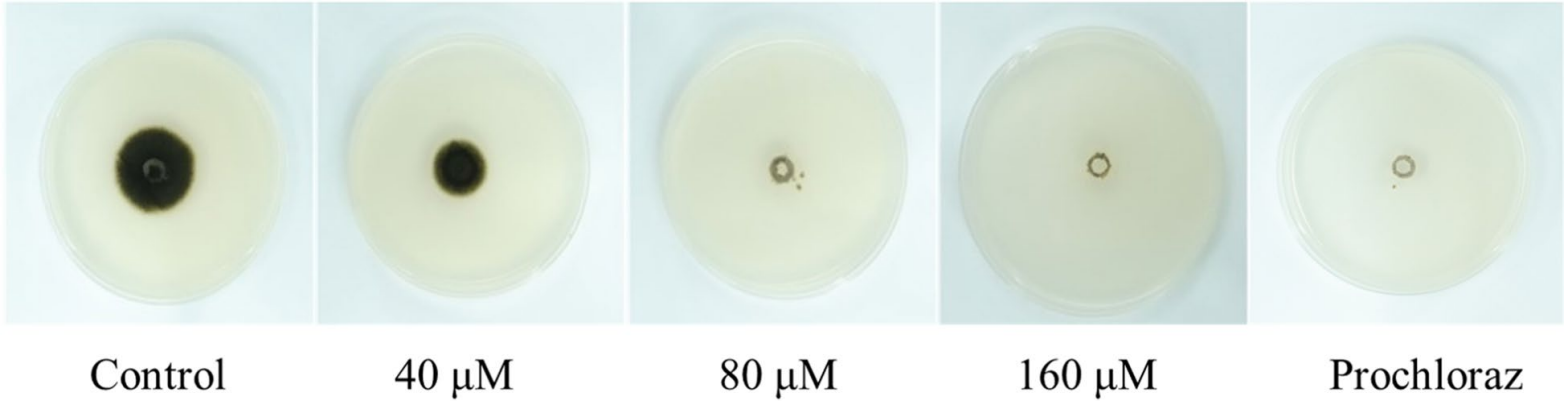
Effect of the growth of *A. brassicicola* by different concentrations of cadmium treatments (0, 40, 80, and 160 μM) in PDA plates

There was a significant difference in the proliferation of *A. brassicicola* with different Cd concentrations (Fig. 1). *A. brassicicola* could grow normally on PDA (Cd-free), but be inhibited on PDA treated with Cd, and the degree of inhibition increased with the increasing of Cd concentration. The growth of *A. brassicicola* is completely inhibited by Cd treatment of 80 μM and above.

#### The elemental defensive effect of Cd to *A. brassicicola* in *B. juncea*

**Fig. 2:**
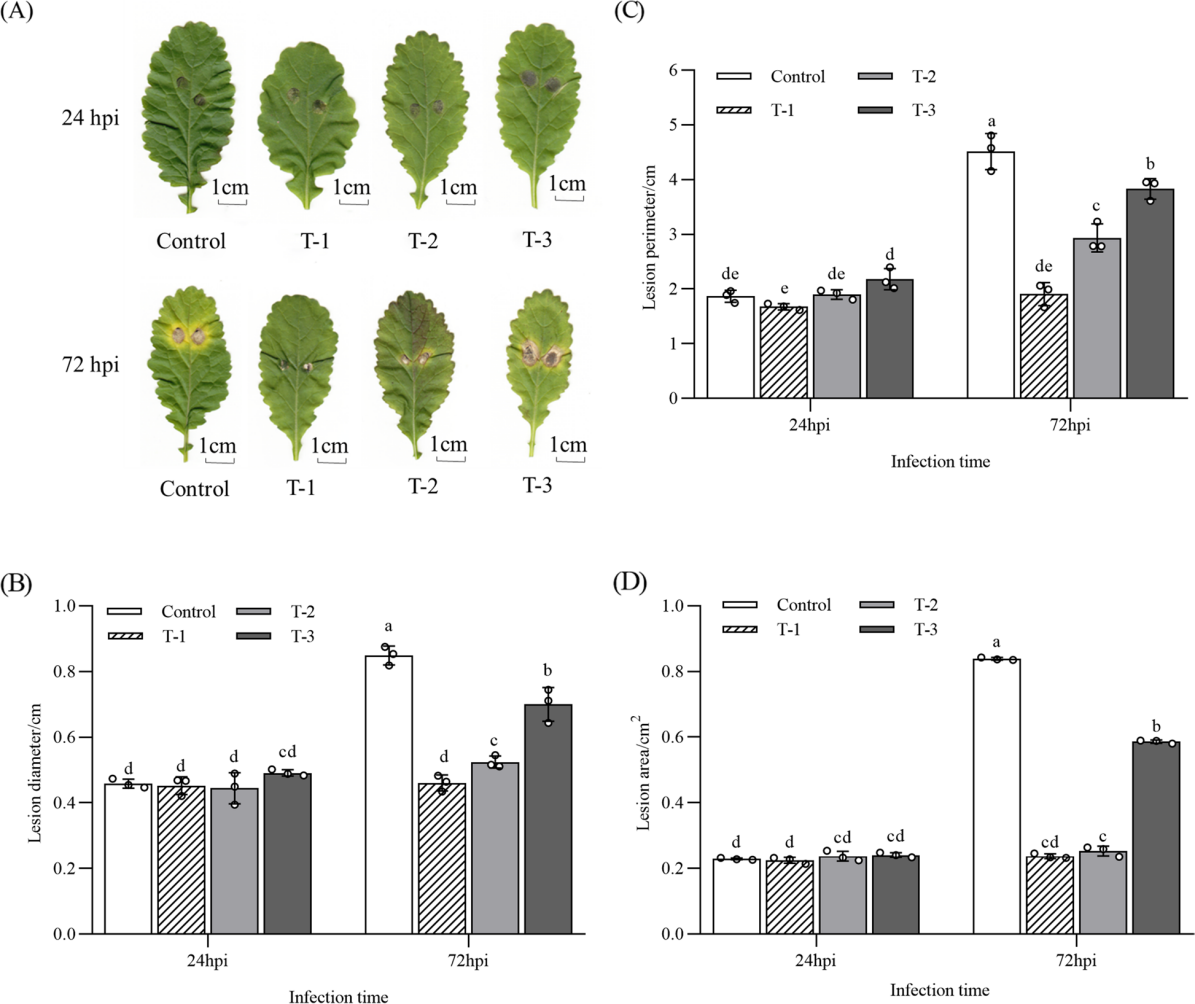
Change of the disease spots at 24 and 72 hpi of *B. juncea* leaves under different concentrations of cadmium treatments. T-1 (180 mg Kg^− 1^), T-2 (360 mg Kg^− 1^), and T-3 (720 mg Kg^− 1^). The control samples were treated only using distilled water under similar conditions. A (disease spots), B (lesion diameter), C (lesion perimeter), and D (lesion area). Values represent the means ± standard deviations of triplicate assays. Values with different letters are significantly different at *P* < 0.05 using LSD test

As shown in Fig. 2, there were no significant differences in the area, perimeter, and diameter of the lesions between the control and the three treatment groups at 24 hpi. The area, perimeter, and diameter of the disease spots at 72 hpi in all treatments were larger than those at 24 hpi. Compared with the control, the lesions on the leaves were smaller in Cd-accumulated *B. juncea* and were negatively correlated with the concentration of Cd. The results indicated that *B. juncea* leaves enriched Cd could produce an elemental defense effect, which could alleviate the disease but not eliminate it.

#### Analysis of spore development in disease spots

**Fig. 3:**
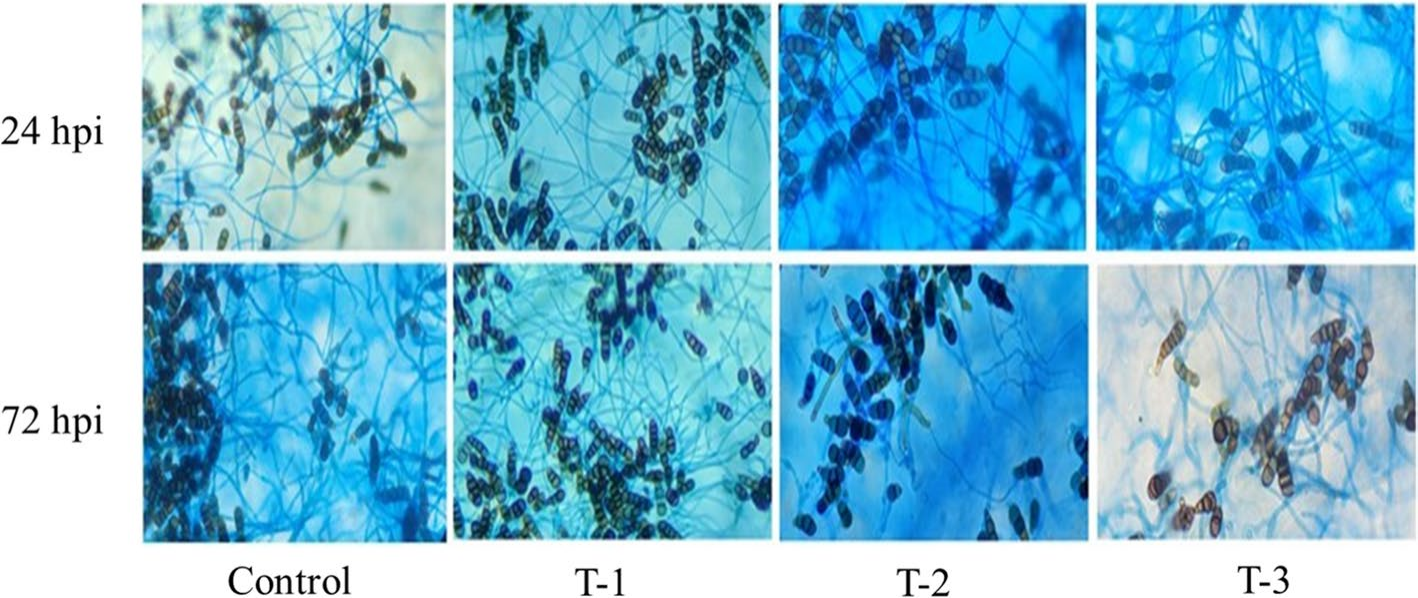
The development *o*f *A. brassicicola* at 24 and 72 hpi under different concentrations of cadmium treatments. T-1 (180 mg Kg^− 1^), T-2 (360 mg Kg^− 1^), and T-3 (720 mg Kg^− 1^). The control samples were treated only using distilled water under similar conditions

There was no obvious change in spore development at 24 hpi in all samples, mainly showing the development degree of bud tubes and the width of hyphae were basically the same without obvious diaphragm. At 72 hpi (Fig. 3), compared with T-1 and T-2 groups, the development of *A. brassicicola* in the control and T-3 were obviously different. It is reflected that the spore bud tube was wider in the control and T-3 than those of T-1 and T-2 treatments, and the mycelium had diaphragms and even distended. Both T-1 and T-2 treatments effectively reduced the spread of disease spots and slowed down the development of the spores of *A. brassicicola*. Hence, the defense effect of T1 treatment was the best. This indicated that the development of *A. brassicicola* was restricted by a certain amount of Cd accumulated in the leaves of *B. juncea*.

### Cd content in *B. juncea* leaves

**Fig. 4:**
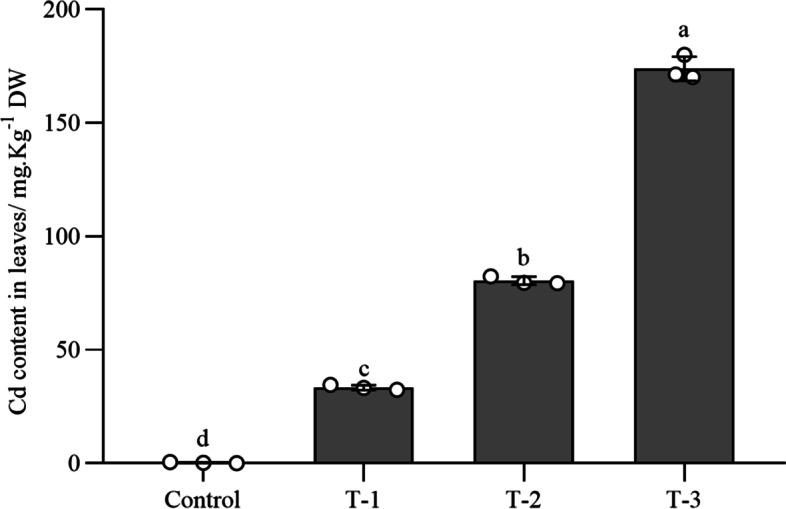
Cd content in *B. juncea* leaves under different concentrations of cadmium treatments. T-1 (180 mg Kg^− 1^), T-2 (360 mg Kg^− 1^), and T-3 (720 mg Kg^− 1^). The control samples were treated only using distilled water under similar conditions. Values represent the means ± standard deviations of triplicate assays. Values with different letters are significantly different at *P* < 0.05 using LSD test

The element defense effect was obvious at 72 hpi, the Cd contents in *B. juncea* leaves of the control, T-1, T-2, and T-3 treatments were 0.29, 33.35, 80.48, and 173.91 mg Kg^− 1^ DW, respectively (Fig. 4). Cd content of leaves in each treatment increased with the increase of Cd concentration in soil. This indicated that there was a positive correlation between Cd contents in *B. juncea* leaves and soil**.**

### SA and JA contents in *B. juncea* leaves

**Fig. 5:**
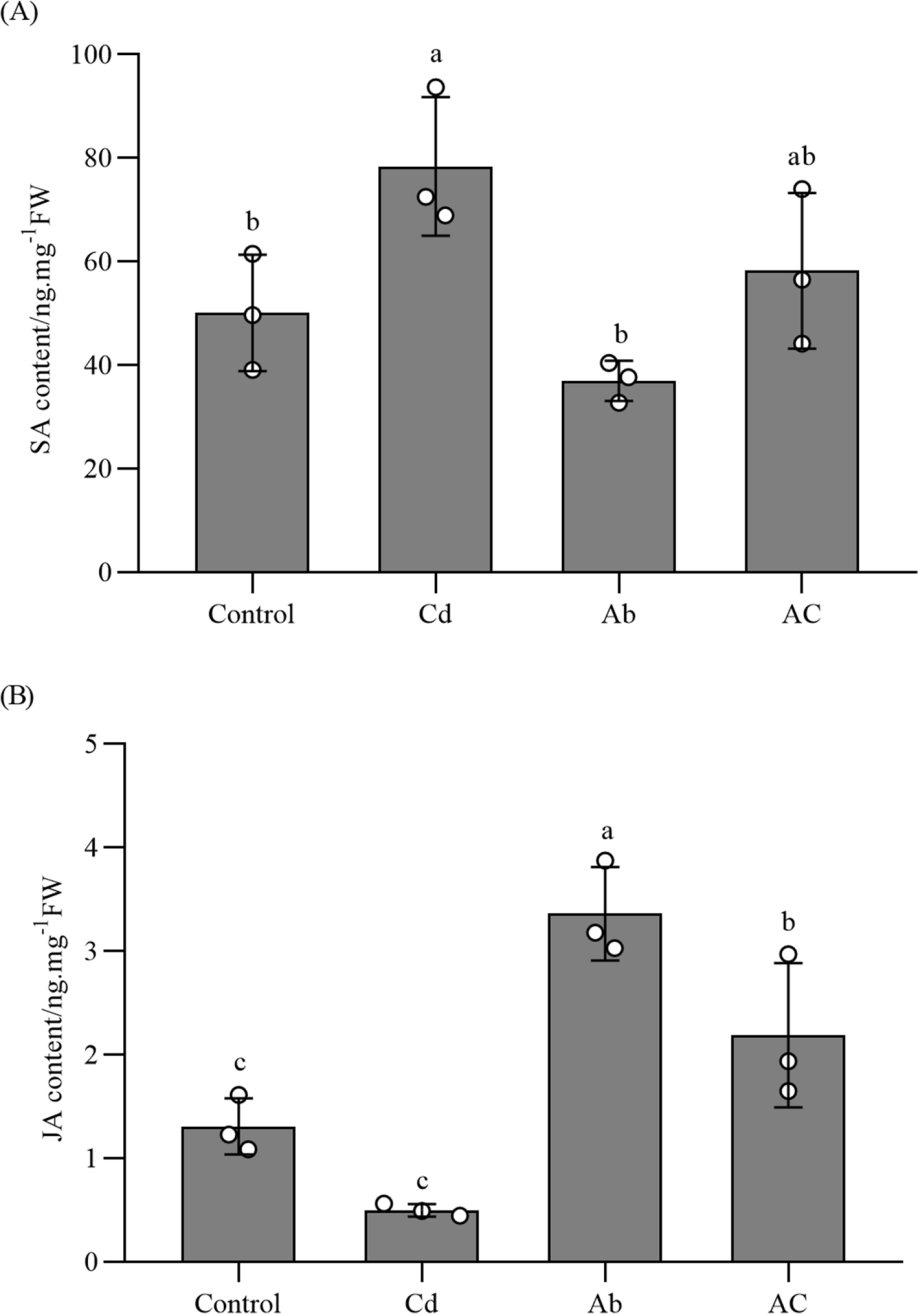
SA (**A**) and JA (**B**) contents in of *B. juncea* leaves in different treatments. Cd, plant treated with cadmium; Ab, plant inoculated with *A. brassicicola*; AC, plant treated with cadmium and inoculated with *A. brassicicola*. The control samples were treated only using distilled water under similar conditions. Values represent the means ± standard deviations of triplicate assays. Values with different letters are significantly different at *P* < 0.05 using LSD test

Compared with the control plants, Cd supply alone resulted in a significant increase of SA contents in leaves (*P* < 0.05), but did not change JA contents (Fig. 5). In comparisons with uninfected plants, whether Cd-stressed or non-Cd-stressed plants, the pathogen could significantly increase JA contents (*P* < 0.05).

### Changes in the expression of defense-related genes in *B. juncea* during the elemental defense

**Fig. 6:**
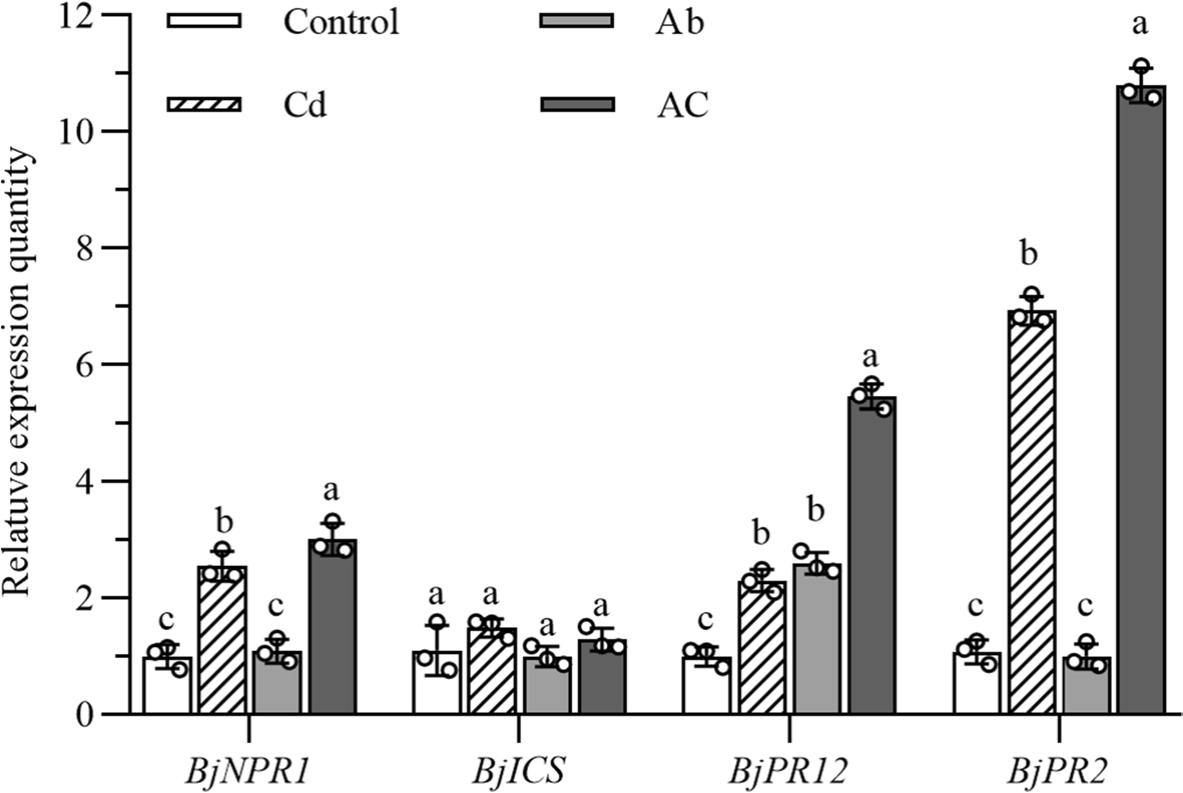
Differential expression of defense-related genes in *B. juncea* under elemental defense. Cd, plant treated with cadmium; Ab, plant inoculated with *A. brassicicola*; AC, plant treated with cadmium and inoculated with *A. brassicicola*. The control samples were treated only using distilled water under similar conditions. Values represent the means ± standard deviations of triplicate assays. Values with different letters are significantly different at *P* < 0.05 using LSD test

As shown in Fig.6, the transcription levels of *BjNPR1*, *BjPR12*, and *BjPR2* in AC treatment were significantly higher than the control, Cd treatment, and Ab treatment (*P* < 0.05). The expression of *BjNPR1* and *BjPR2* was up-regulated in Cd treatment but showed no significant change in Ab treatment*.* Both Cd treatment and Ab treatment resulted in the up-regulated expression of *BjPR12*. However, no significant difference in the expression of *BjICS1* was observed between the control and each treatment.

### The expression of stress-related miRNAs in elemental defense of *B. juncea*

#### Changes of miRNA expression

**Fig. 7:**
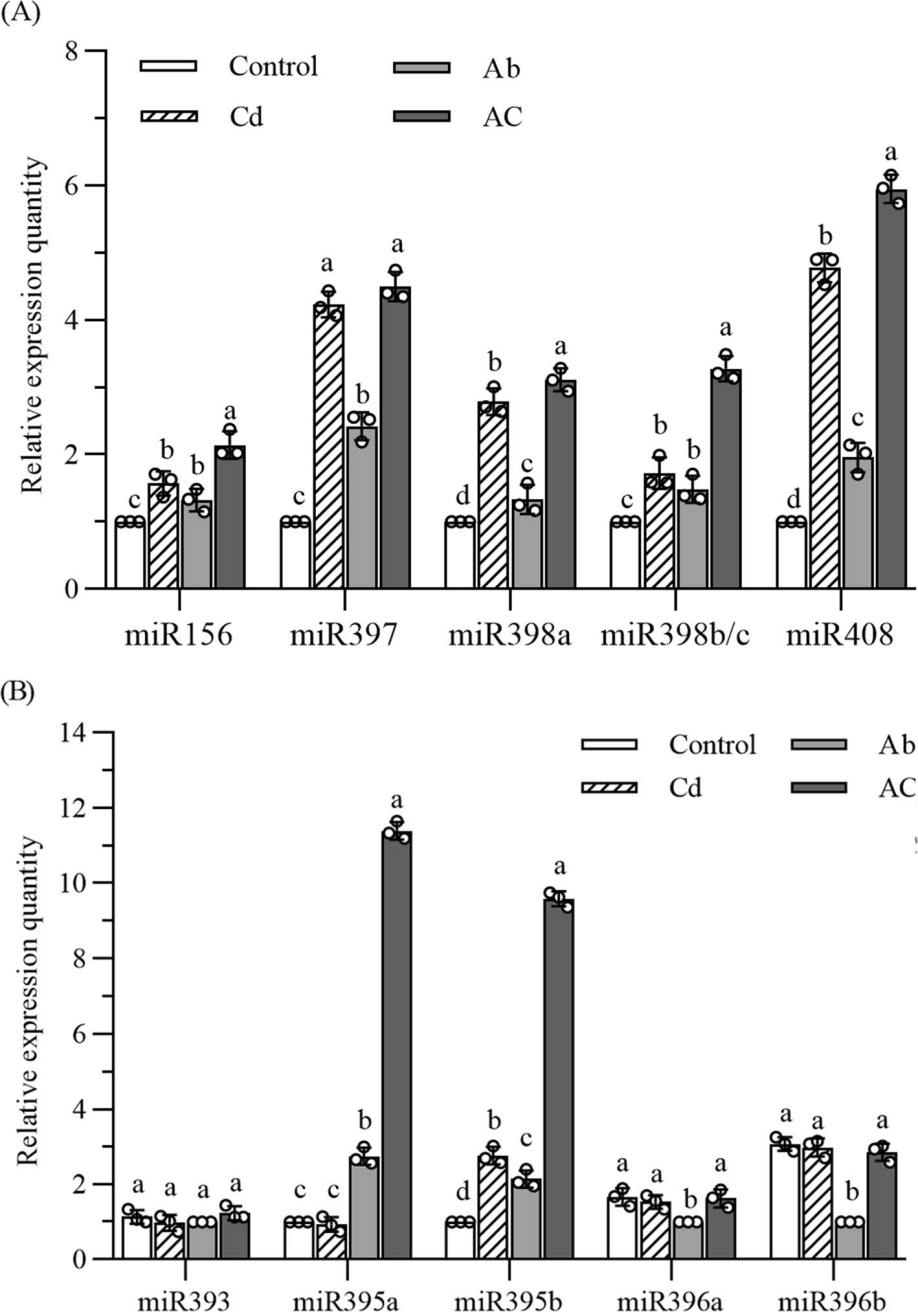
Relative expression of different miRNAs. Cd, plant treated with cadmium; Ab, plant inoculated with *A. brassicicola*; AC, plant treated with Cd and inoculated with *A. brassicicola*. The control samples were treated only using distilled water under similar conditions. Values represent the means ± standard deviations of triplicate assays. Values with different letters are significantly different at *P* < 0.05 using LSD test

As seen from Fig. 7A, the five miRNAs (miR156, miR397, miR398a, miR398b/c, and miR408) related to heavy metal stress response showed similar changes that the expression levels were up-regulated both in Cd treatment and Ab treatment, and further up-regulated in AC treatment. Another five miRNAs (miR393, miR395a, miR395b, miR396a, and miR396b) related to disease resistance presented diverse changes (Fig. 7B). The expression levels of miR393 did not differ significantly among the four groups. The expression level of miR395a was up-regulated in Ab treatment and further up-regulated in AC treatment. However, miR395b was up-regulated in both Cd and Ab treatment and had the highest expression level in AC treatment. The expression levels of miR396a and miR396b were significantly decreased only in Ab treatment (*P* < 0.05).

#### Functional annotation of target genes of the differentially expressed miRNAs

PsRNATarget was used to target match the miRNAs in *B. juncea* transcript data. The results showed that a total of 463 transcription sequences of *B. juncea* were targets of these miRNAs (miR156, miR397, miR398a, miR398b/c, miR408, miR395a, miR395b, miR396a, and miR396b). Through sequence alignment, these target genes were highly similar to those in Chinese cabbage rape (*B. rapa*), European rape (*B. napus*), radish (*Raphanus sativus)*, cabbage (*B. oleracea*), and mustard (*B. juncea*).

Four hundred sixty-three sequences information of *B. juncea* were classified and analyzed according to their functions (Table S1). MiR156 of *B. juncea* mainly targets squamosa promoter binding protein (SPB), oligomeric Golgi complex subunit, rhomboid-like protein, reticulon-like protein, and catalytic enzymes in transcription regulation, metabolite processing modifications. MiR397 mainly targets laccase, E3 ubiquitin ligase, glucanase, and RNA helicase in *B. juncea.* MiR398a and miR398b/c are mainly involved in the transcription and expression of 60S ribosomal proteins and some glycosyltransferase genes. MiR408 mainly targets laccase, L-ascorbate oxidase, and some structural and functional proteins. MiR395a targets ATP sulfurylase, laccase, myosin-binding protein. MiR395b mainly includes key proteins/enzymes such as sulfate transporter, ATP sulfurylase, and serine carboxypeptidase. The target genes of miR396a are involved in various growth-regulating factors and synthesis of biomolecules (pentatricopeptide repeat proteins and RNA helicase). In addition, miR396b can also target serine/threonine phosphatase, blue fluorescent protein, and other protein-coding sequences with catalytic and regulatory functions.

## Discussion

It is reported that some plants can utilize the accumulated metals in their tissues to protect themselves from pathogens, which is an elemental defense effect. There is some evidence to prove the defensive effect. *Arabidopsis helleri* growing on soil accumulated Zn and Cd induced less camalexin after being infected by *Alternaria brassicae*, demonstrating that plants were less stressed by the attack of pathogen [13]. It was shown that nickel hyperaccumulation defends *Streptanthus polygaloides* against pathogens (*Xanthomonas campestris* and *Alternaria brassicicola*) [5]. Selenium hyperaccumulation protected Indian mustard (*Brassica juncea*) from *Alternaria brassicicola and Fusarium* sp. [7]. Similarly, in this study, in comparison with the control, smaller lesions and a lower degree of spore development were observed in T-1, indicating a certain kind of elemental defense in resisting pathogens through the accumulated Cd. In our study, the elemental defense effect was observed in Cd-accumulating *B. juncea* attacked by *A. brassicicola*. The mechanism of the elemental defense effect varies with plant, element, and pathogen. Cd-stressed wheat could produce Cd^2+^-stress associated protein (CSAP) to defend against *Fusarium oxysporum* [22]. When Cd concentration approached the toxicity threshold, it induced a defense signaling pathway, thus enhancing plant response to *Botrytis* attack in *Arabidopsis* [43]. The metal hyperaccumulator plant *Noccaea caerulescens* is mainly protected against Zn-tolerant pathogens by increasing glucosinolates and cell death [43].

Plants invaded by pathogens will develop systemic acquired resistance (SAR) to enhance disease resistance, which is a long-lasting, integrated, broad-spectrum defense measure [44]. Phytohormones, such as salicylic acid (SA), methyl jasmonate (MeJA), ethylene (ET), and abscisic acid (ABA) play vital roles in plant resistance to pathogens attack. SA signaling pathway is involved in the induction of SAR, whereas JA/ET is involved in the activation of induced systemic resistance [45, 46]. After pathogen attack, SA and JA signaling pathways are activated, which further result in the accumulation of PR proteins, thereby minimizing pathogen load or the onset of uninfected plant organs [14]. There is accumulated evidence to suggest that higher levels of the endogenous SA and a series of pathogenesis-related (PR) genes can activate SAR in plants [46]. In our study, SA contents did not show a significant difference between the control and the infected leaves (Fig. 5A), suggesting that defense-related pathways were not effectively activated in this compatible interaction. Conversely, Cd treatment resulted in a significant enhancement in SA contents in leaves. Similar results were observed in Cd- stressed *Noccaea praecox* [20]. It has been reported that exogenous SA protected plants from Cd toxicity by protecting them from Cd-induced oxidative stress in Cd-sensitive plants [22, 47]. In *B. juncea* leaves, JA contents were remarkably enhanced by *A. brassicicola* infection (Fig. 5B).

*NPR1* (non-expressor of pathogen-related gene 1) is considered as a receptor of SA, which can mediate multiple immune responses in plants, particularly the activation of SAR [48]. In addition, *NPR1* plays a cardinal component in inducing defense signaling networks regulated by SA, JA, and ET [49]. SA synthesized from chorismate by means of isochorismate synthase (ICS) plays a pivotal role in plant resistance to pathogens invasion, which is required for *PR1* gene expression and SAR defense responses [50]. In barley, SA was indeed synthesized by an ICS-dependent pathway in response to *Fusarium graminearum* attack, as well as overexpression of *ICS* could increase SA levels [51]. Makandar, et al. [52] reported that the expression of *Arabidopsis thaliana NPR1* gene (*AtNPR1*) enhanced the resistance to *Fusarium graminearum* in wheat. Overexpression of *NPR1* in *B. juncea* and peanut (*Arachis hypogeae* L.) improved the resistances to fungal pathogens [46, 53]. In our study, compared with the control, the expression of *BjNPR1* was up-regulated in Cd treatment and AC treatment, but not Ab treatment. This indicates that *BjNPR1* is involved in the response to Cd, but not to *A. brassicicola* infection.

Overexpression of some PR protein genes, including glucanase (PR2 protein family) and defensin (PR12 protein family), individually or in conjunction greatly improved the level of plant defense response to various pathogens. In the process of plant disease resistance, defensin and glucanase are regarded as molecular markers of JA and SA-mediated SAR, respectively [14]. Overexpression of PR2 genes, such as β-1,3-glucanase and endo-β-1,3(4)-glucanase gene could effectively enhance disease resistance in plants [54–57]. Studies have found that some heavy metals can result in the enhancement of the expression of disease-related proteins in plants. For example, mercuric chloride could cause the obvious expression of chitinases and glucanases in maize leaves [58]. A similar response was observed in our study, in which the expression of *BjPR2* was remarkably upregulated in Cd treatment, but not in Ab treatment, and further up-regulated in AC treatment*.* Some of the PR proteins, known as antimicrobial peptides (AMPs), are a key component of SAR. Plant defensins belong to the PR12 protein family, which exhibit a series of activities, such as antimicrobial and enzyme inhibitory activities, and play crucial roles in plant heavy metal tolerance and development [59]. Many reports have revealed that plant defensins enhanced resistance to many fungal pathogens in radish and rice [60, 61]. Overexpression of plant defensins in transgenic tobacco [62, 63], melon [64], banana [65], and wheat [66] could enhance disease resistance. Additionally, they play significant roles in abiotic stresses such as heavy metals, cold acclimation, wounding, salinity, and drought [67–69]. In this study, the expression of *BjPR12* was up-regulated in Cd and Ab treatments and further up-regulated in AC treatment. This suggests that plants may have some common mechanisms in abiotic stress resistance (represented by heavy metal tolerance) and biotic stress resistance (represented by disease resistance). The Joint Effects Hypothesis states that the elemental and organic defense can coordinate with each other to resist the invasion of pathogens [10, 70]. Our results deemed that *B. juncea* enriched with proper amount of Cd could produce elemental defense effect, as well as enhance SAR. Elemental defense and SAR depending on defense gene expression presented a synergistic effect to defend against *A. brassicicola*.

MiRNAs, such as miR156, miR397, miR398a, miR398b/c, and miR408, are widely involved responses to heavy metal stress in plants. MiR156 is a positive regulator of plant tolerance to Cd stress. The overexpressing miR156 in plants dramatically elevated tolerance to Cd stress. Conversely, plants with lower miR156 expression levels were more sensitive to Cd stress [71]. The expression of miR156a was up-regulated in roots and leaves of *Brassica napus* exposed to Cd [72]. MiR156 of *B. juncea* mainly targets squamosa promoter binding protein (SPB) (Table S1) which plays a vital role in regulating flower and fruit development, gibberellin signaling, and sporogenesis [73]. In addition, miR397 (*Zea mays*) [74], miR398 (*Glycine max*) [75], and miR408 (*B. napus*) [76] were up-regulated when plants were exposed to Cd stress. MiR397 and miR408 of *B. juncea* mainly target laccase (Table S1), which also plays an important role in plant response to environmental stress such as heavy metal, salinity, and drought stress [77, 78]. Huang, et al. [34] reported that miR397a in woody *Citrus* mainly targeted laccase, indirectly affected lignin synthesis, and participated in the adjustment of cell wall structure by regulating laccase genes expression to resist boron toxicity. The miR398 family was mainly involved in response to drought, biological and light stress [37]. Similar to the results of previous studies, the expression levels of these miRNAs (miR156, miR397, miR398a, miR398b/c, and miR408) were all up-regulated under Cd treatment in *B. juncea* up-regulated at varying degrees. These miRNAs were also up-regulated after *A. brassicicola* infection, indicating that they were also involved in disease resistance. These miRNAs all had the highest expression levels in the AC treatment, suggesting that the expression levels of these miRNAs were further increased in response to the combined stress of Cd and pathogen in *B. juncea*.

In addition, miRNA-mediated defense responses can effectively regulate plant gene expression, thus making the overall physiological state of the plant more suitable for the needs of disease resistance [75]. It is found that the miR393 family of plants had different spatiotemporal expression models in response to various environments, and they participated in the regulatory network of stress response by targeting TIR1 (transport inhibitor response 1, TIR1), such as auxin signal transduction pathway [79]. When *Arabidopsis* was infected with pathogen-related molecules such as Flg22 (a 22-amino acid peptide at the N-terminal of eubacterial flagellin), miR393 could be up-regulated to inhibit the expression of F-box protein. In this way, the growth of pathogens was restricted by regulating the auxin signal transducing pathway in *Arabidopsis* [80]. However, the results of this experiment showed that miR393 was not involved in the response to Cd stress and *A. brassicicola* infection in *B. juncea*. This may be because the time for miR393 to respond to *A. brassicicola* infection was not at 72 hpi in *B. juncea*.

Up-regulated miR395 could target and regulate WRKY26 to participate in the defense of apple (*Malus domestica*) against the invasion of *A.alternaria* [81]*.* Also, Zhang, et al. [82] reported that miR395 would participate in Cd detoxification in transgenic rapeseed (*Brassica napus*). In the present study, miR395a did not respond to Cd stress but responded to *A. brassicicola* infection. While miR395b responded to both Cd tress and *A. brassicicola* infection. Both miR395a and miR395b had the highest expression levels in AC treatment, indicating that the expression of miR395 was further enhanced in response to the combined stress of Cd and pathogen in *B. juncea*.

In *Arabidopsis thaliana* infected with *Plectosphaerella cucumerina* spores, the transcription levels of miR396 precursors and mature miR396 were down-regulated, while growth-regulating factors (GRF) targeted by miR396 were generally upregulated [40]. In our experiment, after the infection of *A. brassicicola* in *B. juncea*, the expression levels of miR396a and miR396b were down-regulated. However, there was no significant change in the expression level of miR396a and miR396b under the combined treatment of Cd and *A. brassicicola*. This may be because the expression pattern of miR396 is affected by heavy metal enrichment and disease resistance, which is different from the response mode of heavy metal stress or pathogen invasion alone. This implies that since Cd plays a defensive role, miR396 is not required for the response to pathogen infection, which exemplifies a trade-off between elemental defense and organic defense.

## Conclusions

As stated above, *B. juncea* displayed an obvious elemental defense effect under a certain concentration of Cd stress when challenged with *A. brassicicola* infection. Some phytohormones (JA and SA), defense-related genes (*BjNPR1*, *BjPR12*, and *BjPR2*), and miRNAs (miR156, miR397, miR398a, miR398b/c, miR408, miR395a, miR395b, miR396a, and miR396b) might play essential roles during elemental defense in Cd-stressed *B. juncea*. Consequently, our findings provide strong support to the elemental defense hypothesis in Cd-stressed *B. juncea*.

## Materials and methods

### Plant materials and growth conditions

Seeds of *B. juncea* ‘Purple-leaf Mustard’ acquired from Yan et al. [83] were surface sterilized and germinated in Petri dishes with deionized water, then planted individually in pots (10 × 8.5 × 7 cm) contained mixed soil (peat: vermiculite = 3:1). The plants were grown in the climate house with a daytime temperature of 25 °C and a night-time temperature of 23 °C for 16 h day/8 h dark photoperiod (light intensity 200 μmol·m^− 2^ s^− 1^).

### Pathogen growth and preparation of the conidia suspension

The pathogen *A. brassicicola* (strain ACCC 37296) was obtained from the Agricultural Culture Collection of China (ACCC). The pathogen was grown in potato dextrose agar (PDA) medium at 28 °C in dark. After 7 days, the mycelium at the culture surface was flushed with 10 mL of 5‰ (*v/v*) Tween-20, then filtered with a 4-layer sterile gauze to collect the suspension. The conidia suspension with 2.5 × 10^6^ spores per milliliter using a hemocytometer was prepared for infecting seedlings of *B. juncea*.

### Infection assays

When the seedlings were 14-days old, CdCl_2_ 2.5H_2_O solutions were added to mixed soil to final concentrations of 0 mg Kg^− 1^ (control), 180 mg Kg^− 1^ (T-1), 360 mg Kg^− 1^ (T-2), and 720 mg Kg^− 1^ (T-3), respectively. The Cd concentrations were selected according to the report of Zhang, et al. [84] After 14 days, the 28-days old plants were used for subsequent experiments.

The young leaves of 28-days old plants were inoculated with the conidia suspension of *A. brassicicola* according to the method reported by Mandal, et al. [85] with slight modifications. The fourth true leaf of seedlings with similar size was selected as the inoculation leaves. A total of 20 μL of suspension of 2.5 × 10^6^ conidial spores per mL were inoculated equally on two sides of the midrib at the leaf surface. The inoculated seedlings were cultured in a chamber at 28 °C with 90% relative humidity.

### Analysis of *A. brassicicola* development

Development of the *A. brassicicola* was determined using the trypan blue staining technique according to described by Koch and Slusarenko’ [86] with slight modifications. The leaves at 12, 24, 48, and 72 hpi were immersed in lactophenol-trypan blue solution (containing 10 mL of lactic acid, 10 mL of glycerol, 10 g of phenol, 10 mg of trypan blue, and 10 mL of distilled water), and then boiled for 1 min, finally decolorized in bleaching liquid (containing 25 g of chloral hydrate and 10 mL of distilled water) for 12 h. The decolorized leaves were soaked in 50% glycerin for preservation and optical microscopic observation. Lesions on *B. juncea* leaves were immediately scanned with a CanoScan LiDE scanner (Canon, Japan). Parameters (diameter, perimeter, and area) of lesions were assayed using Image J software. Three biological replicates were performed for each treatment.

### Analysis of in vitro toxicity of Cd to *A. brassicicola*

The toxicity of Cd to *A. brassicicola* was analyzed by the colony diameter method [87]. A disc (7 mm) of fully grown fungi (at 28 °C for 1 week) was inoculated on the center of PDA plates with different Cd concentrations (0-negative control, 40, 80, 160 μM) at 28 °C for 7 d, respectively. In addition, 400 mg/L 50% Prochloraz was added into the PDA medium inoculated *A. brassicicola* as a positive control. The plates were cultured at 28 °C for 7 d to observe the inhibition of the fungi growth. Three biological replicates were performed for each treatment.

### Determination of Cd content

*B. juncea* leaves were digested based on the method of Jiang, et al. [88], and the Cd content was assayed by iCE 3000 Series AA Spectrometers (Thermo Scientific, USA) with three biological replicates. The Cd standard curve (Abs = 0.46900C + 0.019858, R^2^ = 0.997) was plotted using the Cd standard solution (China national standard sample no. GSB04–1721-2004).

### Measurements of SA and JA contents in *B. juncea* leaves

Four experimental groups were used for measurements of SA and JA contents: (1) Cd group (leaves of 31-days old plants which had been treated with 180 mg Kg^− 1^ CdCl_2_ 2.5H_2_O for 17 days), (2) Ab group (leaves of 31-days old plants which had been inoculated with *A. brassicicola* for 72 h), (3) AC group (leaves of 31-days old plants which had been treated with 180 mg Kg^− 1^ CdCl_2_ 2.5H_2_O for 17 days and inoculated with *A. brassicicola* for 72 h), and (4) control group (leaves of 31-days old plants treated only using distilled water under similar conditions). The extraction, purification, and chromatographic analytical procedure of SA and JA were performed according to the method of Zhou, et al. [89]. Contents of SA and JA in *B. juncea* leaves were assayed using liquid chromatography-tandem mass spectrometry (8030 plus, Shimadzu, Japan) based on MS conditions as reported by Zhou, et al. [89]. The analysis was performed with three biological replicates for each treatment.

### Quantitative real-time polymerase chain reaction (qRT-PCR) assays

Based on the method described by Nayanakantha, et al. [16], leaves of Cd、Ab、AC, and control groups described above were used for RNA extraction. Total RNAs were extracted from samples of each line using RNA Isolation Kit (TIANMO, BEIJING). Absorbances at wavelengths (230, 260, and 280 nm) were monitored by BioPhotometer D30 (Eppendorf, Germany) and agarose gel examination was performed to determine RNA integrity. cDNA was synthesized from total RNA by Oligo dT (18 T) and Random 6-mers primers (Accurate Biology, Hunan, China). Changes in the expression of four defense-related genes, *BjNPR1* (GenBank accession NO. DQ359129) [46], *BjPR12* (GenBank accession NO. DQ191752) [69], *BjPR2* (GenBank accession NO. DQ359125) [90], and *BjICS1* (a highly similar sequence to *Arabidopsis ICS1*, GenBank accession NO. AY056055) in the *B. juncea* transcriptome, not published) [50], were analyzed in this study. cDNAs coding miRNAs were synthesized from total RNA and primed with specific stem-loop reverse transcriptional primer, expression changes of these miRNAs were amplified with a specific forward primer and a universal revise primer. Primers used in this study were listed in Tables S2 and S3.

The total reaction volume is 20 μL, including10 μL of 2 × SYBR® Green Pro Taq HS Premix with ROX, 1 μL of each primer, 2 μL of cDNA template, and 6 μL of PCR-grade water. PCR conditions included a hold stage initially at 50 °C for 2 min and further 95 °C for 30 s, followed by 35 cycles of 95 °C for 5 s, 60 °C for 30 s before the final melt curve stage. Three biological replicates were set for each treatment, and three technical replicates were set for each biological replicate. *Actin* (GenBank accession NO. NM_001315560.1) was used as the reference gene to calculate the relative expression levels of defense-related genes and miRNAs in different samples by the comparative Ct method [91].

### Target gene prediction, functional annotation, and analysis of differentially expressed miRNA

Differentially expressed miRNAs are selected to predict their target genes using psRNATarget program (http://plantgrn.noble.org/psRNATarget/analysis?function=3). Function annotation and classification analysis of the target sequences of miRNAs according to the online database Web BLAST (https://blast.ncbi.nlm.nih.gov/Blast.cgi).

### Data analysis

SPSS Statistics 25 software was used for heterogeneity of variance of ANOVA test, followed by single-factor ANOVA analysis and significant difference calculation. Origin 9.1 was used for plotting statistical graphs. The differences at *P* < 0.05 were shown significant using the LSD test. Three biological replicates were performed for each treatment.

## Supplementary Information

**Additional file 1: Table S1.** Functional annotation and classification of miRNA target genes.

**Additional file 2: Table S2.** Specific stem-loop RT primers for miRNAs.

**Additional file 3: Table S3.** Primers for expression detection of defense-related genes.

**Additional file 4: Figure S1.** Symptoms and lesion parameters of *B. juncea* leaves infected by *A. brassicicola* at different inoculation time (12, 24, 48, and 72 hpi). A (disease spots), B (lesion diameter), C (lesion perimeter), and D (lesion area). Values represent the means ± standard deviations of triplicate assays. Values with different letters are significantly different at *P* < 0.05 using LSD test.

**Additional file 5: Figure S2.** The development of *B. juncea* infected by *A. brassicicola* at different inoculation time (12, 24, 48, and 72 hpi).

## Data Availability

The datasets used and/or analyzed during the current study available from the corresponding author on reasonable request.
