# Supplementary material for: The elemental defense effect of cadmium on Alternaria brassicicola in Brassica juncea
Source: BMC Plant Biol. 2022 Jan 5;22:17. doi: 10.1186/s12870-021-03398-4 (PMC8729108; doi:10.1186/s12870-021-03398-4)
Supplement: Supplementary file 2 — Additional file 2: Table S2. Specific stem-loop RT primers for miRNAs. [file 12870_2021_3398_MOESM2_ESM.docx]

**Table S2.** Specific stem-loop RT primers for miRNAs

| Primer name | Sequence (5’→ 3’) |
| --- | --- |
| RT-miR156 | GTCGTATCCAGTGCAGGGTCCGAGGTATTCGCACTGGATACGACGTGCTC |
| RT-miR393 | GTCGTATCCAGTGCAGGGTCCGAGGTATTCGCACTGGATACGACGGATCAA |
| RT-miR395a | GTCGTATCCAGTGCAGGGTCCGAGGTATTCGCACTGGATACGACGAGTTC |
| RT-miR395b | GTCGTATCCAGTGCAGGGTCCGAGGTATTCGCACTGGATACGACGAGTCC |
| RT-miR396a | GTCGTATCCAGTGCAGGGTCCGAGGTATTCGCACTGGATACGACAAGTTC |
| RT-miR396b | GTCGTATCCAGTGCAGGGTCCGAGGTATTCGCACTGGATACGACCAGTTC |
| RT-miR397 | GTCGTATCCAGTGCAGGGTCCGAGGTATTCGCACTGGATACGACACATCA |
| RT-miR398a | GTCGTATCCAGTGCAGGGTCCGAGGTATTCGCACTGGATACGACCAGGGG |
| RT-miR398b | GTCGTATCCAGTGCAGGGTCCGAGGTATTCGCACTGGATACGACCAGGGG |
| RT-miR408 | GTCGTATCCAGTGCAGGGTCCGAGGTATTCGCACTGGATACGACAGCCAG |
