# Supplementary material for: The elemental defense effect of cadmium on Alternaria brassicicola in Brassica juncea
Source: BMC Plant Biol. 2022 Jan 5;22:17. doi: 10.1186/s12870-021-03398-4 (PMC8729108; doi:10.1186/s12870-021-03398-4)
Supplement: Supplementary file 3 — Additional file 3: Table S3. Primers for expression detection of defense-related genes. [file 12870_2021_3398_MOESM3_ESM.docx]

**Table S3.** Primers for expression detection of defense-related genes

| Target gene | Forward primer (5’→ 3’) | Revise primer (5’→ 3’) |
| --- | --- | --- |
| *BjNPR1* | AGGAGCCGAGTTTGATAGCG | CAGGGCAAGACAGGCAGAAG |
| *BjICS1* | AGATTTGTTGTTGCCGTTGC | CTATGCGGGGACAGGGAT |
| *BjPR12* | CCATCATCACCCTTATCTTCCT | ACAACTTCTGACCGTCCACCAT |
| *BjPR2* | GTCTGGGGTTAGGGTGTTAGC | ACTTCCGATGGATTTGGTAGG |
| *Actin* | GAATCCACGAGACGACTTACAAC | CGATCCAGACACTGTACTTCCTC |
| miR156 | GCGGCCGGTGACAGAAGAGAGT | GTGCAGGGTCCGAGGT |
| miR393 | GGCTTGTCCAAAGGGATCGCA |  |
| miR395a | CGGCGTGCTGAAGTGTTTGGAG |  |
| miR395b | GGCGTGCTGAAGTGTTTGGGG |  |
| miR396a | GCGGCGGTTCCACAGCTTTCTT |  |
| miR396b | GCGGCGGTTCCACAGCTTTCTT |  |
| miR397 | GCGGCGGTCATTGAGTGCAGCGT |  |
| miR398a | GCGGCGGTGTGTTCTCAGGTCA |  |
| miR398b | GCGGCGGTGTGTTCTCAGGTCA |  |
| miR408 | GCGGCCGTGCACTGCCTCTTCC |  |
