# Supplementary figures and images for: The elemental defense effect of cadmium on Alternaria brassicicola in Brassica juncea
Source: BMC Plant Biol. 2022 Jan 5;22:17. doi: 10.1186/s12870-021-03398-4 (PMC8729108; doi:10.1186/s12870-021-03398-4)

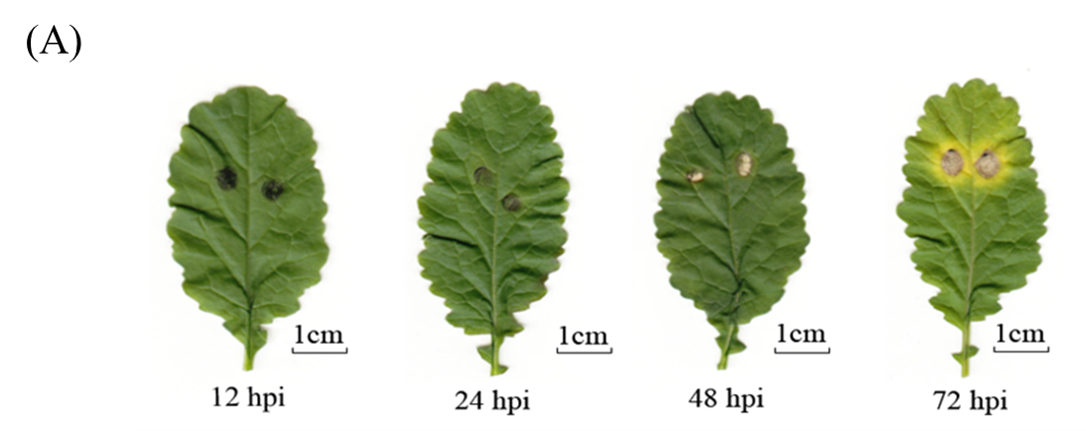

Supplement: Supplementary file 4 — Additional file 4: Figure S1. Symptoms and lesion parameters of B. juncea leaves infected by A. brassicicola at different inoculation time (12, 24, 48, and 72 hpi). A (disease spots), B (lesion diameter), C (lesion perimeter), and D (lesion area). Values represent the means ± standard deviations of triplicate assays. Values with different letters are significantly different at P < 0.05 using LSD test. [file 12870_2021_3398_MOESM4_ESM.zip › Fig.S1A.tif]

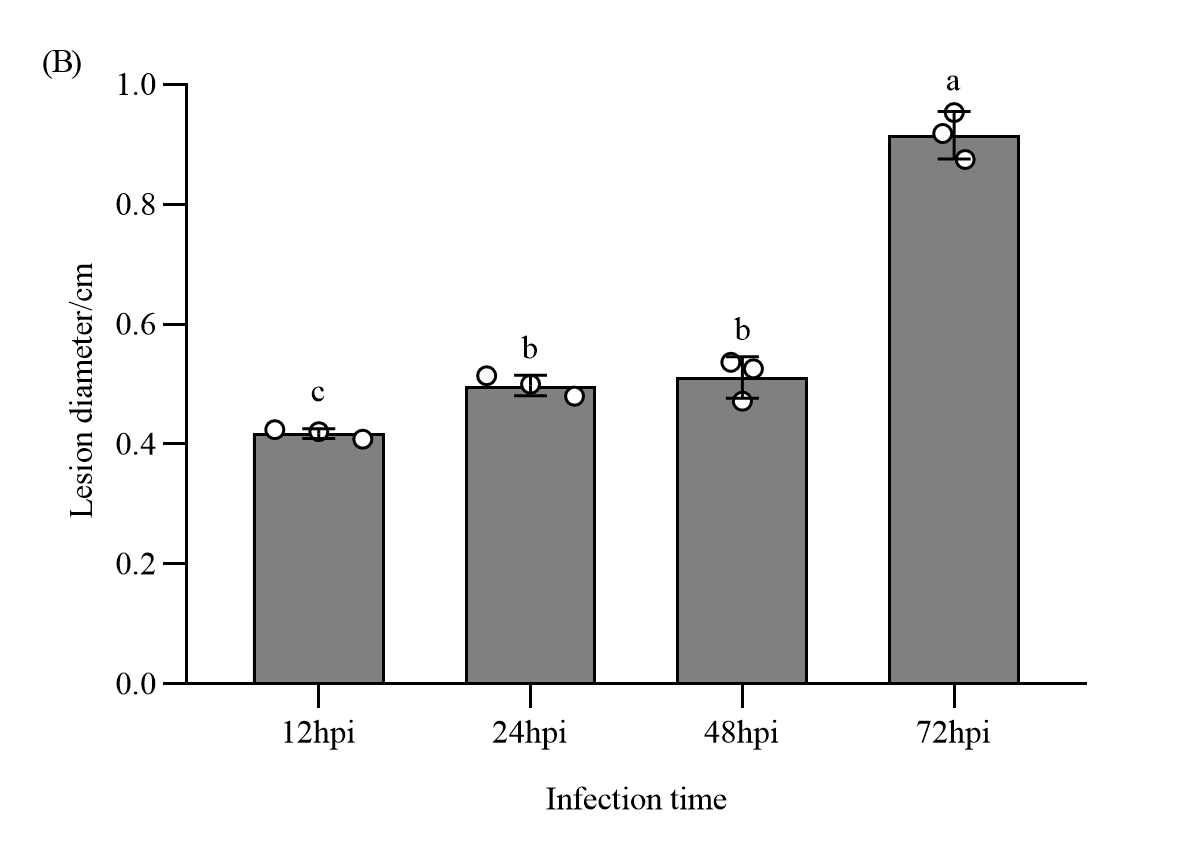

Supplement: Supplementary file 4 — Additional file 4: Figure S1. Symptoms and lesion parameters of B. juncea leaves infected by A. brassicicola at different inoculation time (12, 24, 48, and 72 hpi). A (disease spots), B (lesion diameter), C (lesion perimeter), and D (lesion area). Values represent the means ± standard deviations of triplicate assays. Values with different letters are significantly different at P < 0.05 using LSD test. [file 12870_2021_3398_MOESM4_ESM.zip › Fig.S1B.tif]

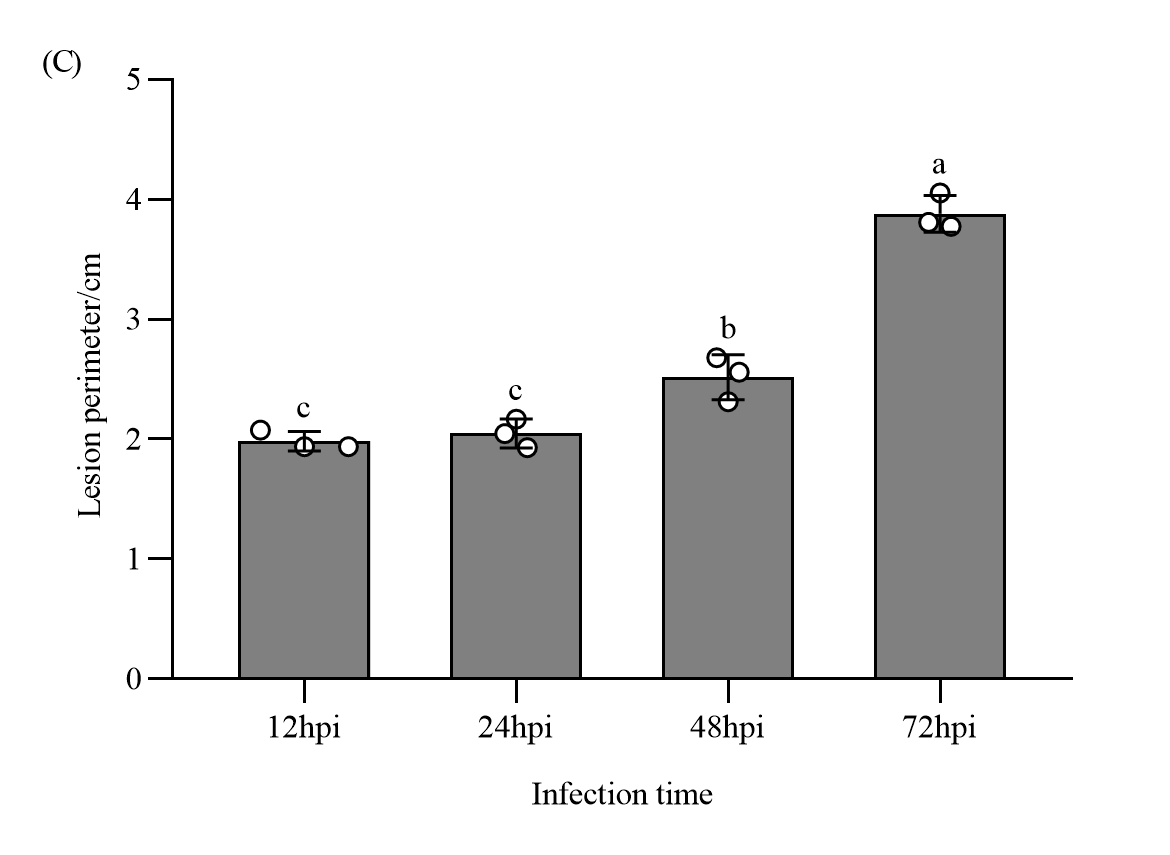

Supplement: Supplementary file 4 — Additional file 4: Figure S1. Symptoms and lesion parameters of B. juncea leaves infected by A. brassicicola at different inoculation time (12, 24, 48, and 72 hpi). A (disease spots), B (lesion diameter), C (lesion perimeter), and D (lesion area). Values represent the means ± standard deviations of triplicate assays. Values with different letters are significantly different at P < 0.05 using LSD test. [file 12870_2021_3398_MOESM4_ESM.zip › Fig.S1C.tif]

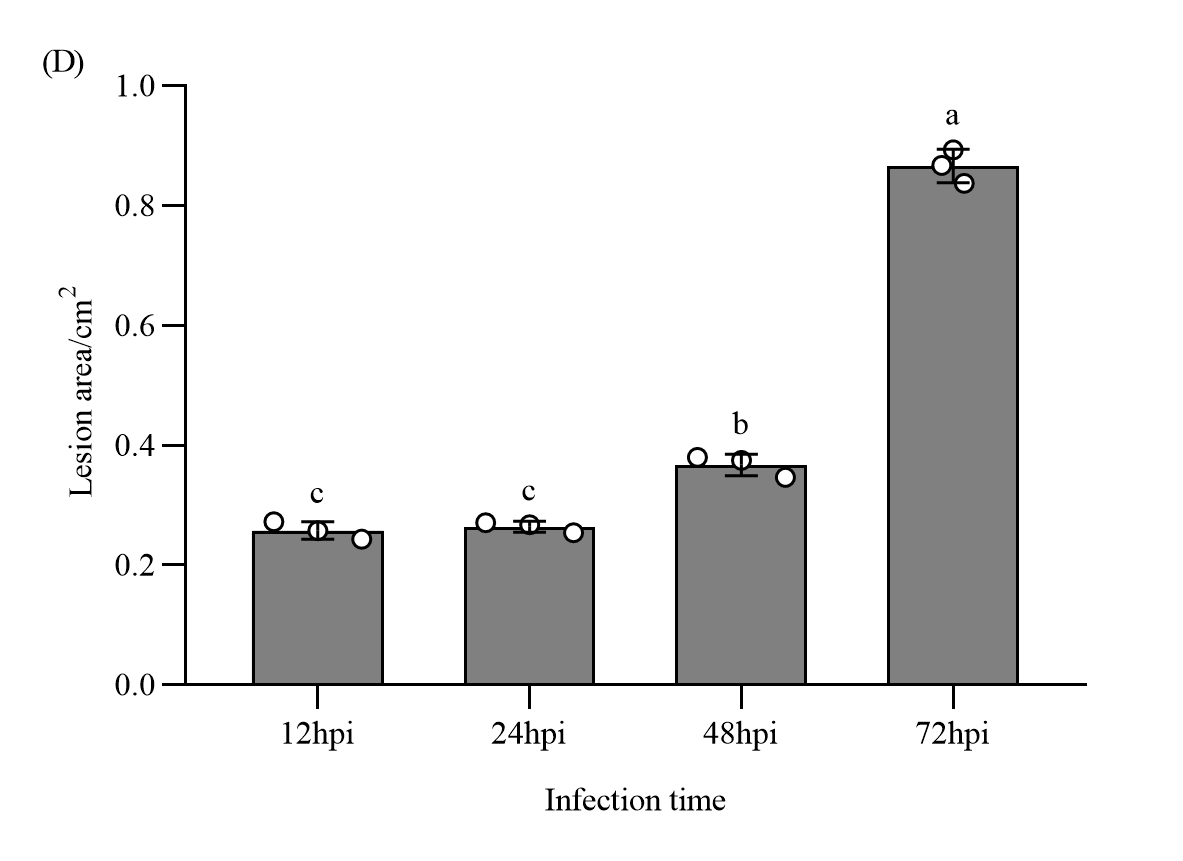

Supplement: Supplementary file 4 — Additional file 4: Figure S1. Symptoms and lesion parameters of B. juncea leaves infected by A. brassicicola at different inoculation time (12, 24, 48, and 72 hpi). A (disease spots), B (lesion diameter), C (lesion perimeter), and D (lesion area). Values represent the means ± standard deviations of triplicate assays. Values with different letters are significantly different at P < 0.05 using LSD test. [file 12870_2021_3398_MOESM4_ESM.zip › Fig.S1D.tif]

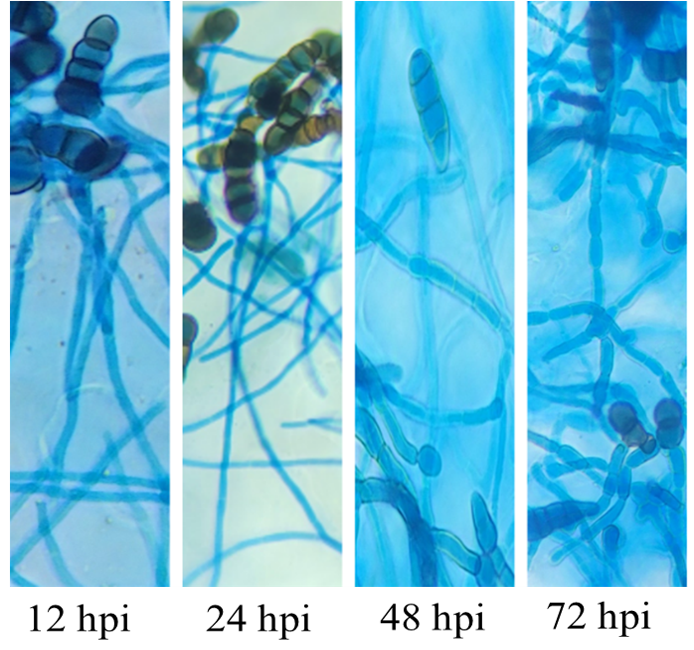

Supplement: Supplementary file 5 — Additional file 5: Figure S2. The development of B. juncea infected by A. brassicicola at different inoculation time (12, 24, 48, and 72 hpi). [file 12870_2021_3398_MOESM5_ESM.tif]
